# Metastatic rectal cancer in the ampulla of Vater: A unique case

**DOI:** 10.1002/cnr2.1510

**Published:** 2021-07-17

**Authors:** Athina A. Samara, Ioanna‐Konstantina Sgantzou, Alexandros Diamantis, Alexandros Kokkalis, Konstantinos Tsapakidis, Maria Tolia, Gregory Christodoulidis, Christos Rountas, Dimitris Zacharoulis

**Affiliations:** ^1^ Department of Surgery University Hospital of Larissa Larissa Greece; ^2^ Department of Radiology University Hospital of Larissa Larissa Greece; ^3^ Department of Oncology University Hospital of Larissa Larissa Greece; ^4^ Department of Radiotherapy University of Crete Heraklion Greece

**Keywords:** immunochemistry, metastatic, rectal cancer, secondary, Vater

## Abstract

**Background:**

A metastatic lesion located in the ampulla of Vater is considered extremely rare, with only 32 cases reported globally.

**Case:**

A 65‐year‐old patient was primarily diagnosed with a rectal adenocarcinoma. Twenty‐four months later as part of the oncological follow‐up, the patient was diagnosed with a single secondary tumor in the ampulla of Vater. After undergoing a pancreaticoduodenectomy (Whipple procedure), the patient experienced an uneventful recovery and received adjuvant chemotherapy. Sixteen months later the patient remained disease‐free.

**Conclusion:**

To the best of our knowledge, the present case represents the first reported metastatic tumor in the ampulla of Vater, originating from a rectal adenocarcinoma. This case underlines the critical role of immunohistochemistry in arriving at a correct diagnosis in order to guide clinical decision‐making.

## 1 INTRODUCTION

Colorectal cancer is the third most common cancer worldwide, with an incidence ranging between 0.4% and 3.6% in Europe.^1^, ^2^ Despite favorable oncological results through the introduction of total mesorectal and mesocolic excision, as well as the introduction of neoadjuvant chemo and radiotherapy, the incidence of local recurrence still ranges from 4% to 8%, with distant recurrences predominating.^3^, ^4^

Typical sites of metastatic rectal cancer include the liver, lungs, peritoneum, retroperitoneally, and lymph nodes.^4^ Metastatic disease located in the small intestine is rare and primarily originates from melanoma, breast or lung cancer.^5^ In order to differentiate another primary malignancy from a recurrence of rectal cancer located in the small intestine, a detailed pathological and immunohistochemical study is required.^6^ Specifically, a metastatic lesion to the ampulla of Vater is considered extremely rare, with only 32 cases reported in the literature.^7^

To the best of our knowledge, we present a unique clinical case of a patient with a single recurrent tumor from rectal cancer, located in the ampulla of Vater.

## 2 CASE PRESENTATION

**FIGURE 1:**
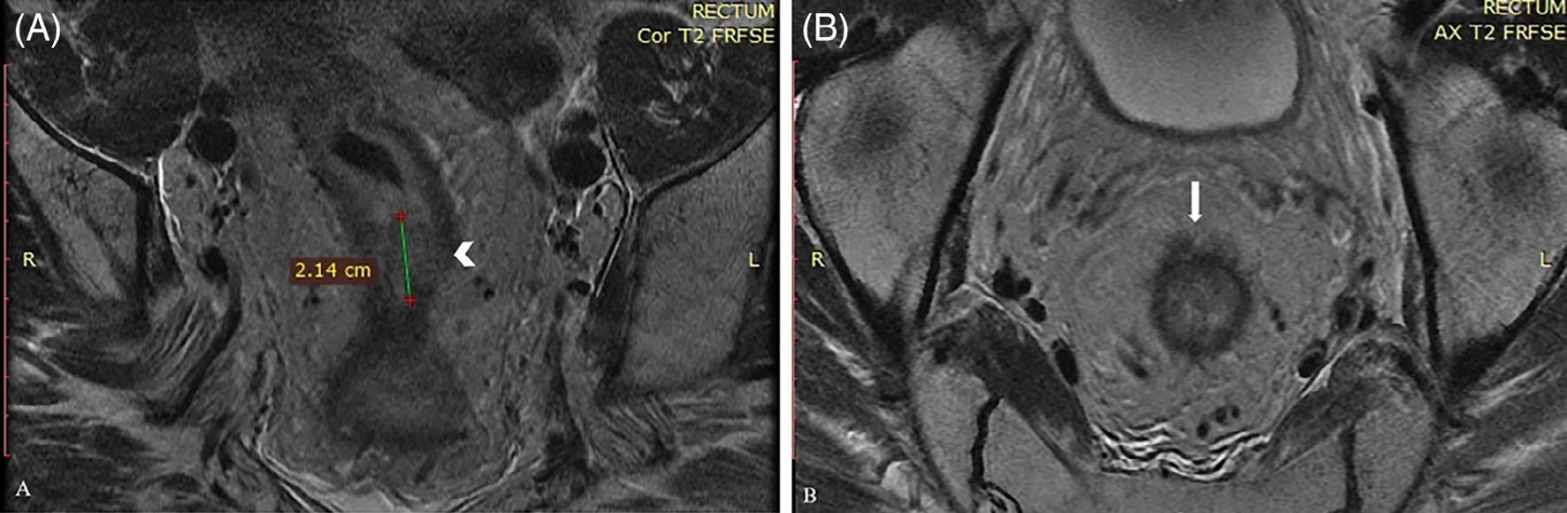
(A) After Neoadjuvant Radiotherapy Magnetic Resonance Imaging (MRI) (Protocol for rectal cancer). Coronal T2 sequence. A soft tissue (arrowhead) measuring 2.1 cm. (B) After Neoadjuvant Radiotherapy MRI (Protocol for rectal cancer). Axial T2, local blurring (arrow) of the perirectal tissue (extends <5 mm beyond muscularis propria, T3b). Mesorectal fascia and levetor ani muscles without signs of infiltration. Extramural Vascular Invasion (−). Lymph nodes without pathological findings

A 65‐year‐old patient with an unremarkable medical history was primarily diagnosed with a stage III rectal adenocarcinoma. A multidisciplinary team determined that the patient should undergo neoadjuvant chemotherapy (capecitabine) and radiotherapy, followed by surgical resection. Eventually, after the downstaging of the tumor (Figure 1), the patient underwent a low anterior resection (pT3N0) and experienced an uneventful recovery. The histopathological results revealed an intestinal adenocarcinoma with negative margins (R0 resection),with negative lymph nodes and without vascular and perineural invasion. The immunohistochemical study was CK7 (−), CD20 (+), CDX2 (+) and DPC4 (+). Four weeks after the resection the patient received nine cycles of adjuvant chemotherapy with oxaliplatin and fluoropyrimidine (FOLFOX) in order to complete 6 months of perioperative treatment according to NCCN guidelines for rectal cancer.^8^ Following this treatment, regular oncological follow‐up occurred with biannual biochemistry and Computer Tomography (CT) examinations.

**FIGURE 2:**
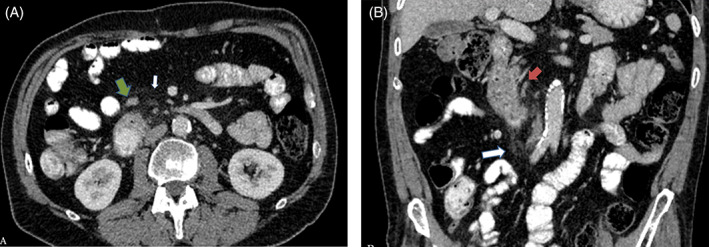
(A) Multidetector computed tomography (MDCT) with intravenous contrast in portal phase A. Axial. Periduodenal fat stranding (white arrow) and dilated local lymph nodes (green arrow). and (B) MDCT with intravenous contrast in portal phase B. Coronal. Periduodenal fat stranding (white arrow) and dilated local lymph nodes (green arrow), with the largest one (red arrow) measuring 7 mm

Twenty‐four months later as part of the oncological follow‐up, the patient underwent an abdominal CT scan where a suspicious wall thickening was revealed in the second portion of the duodenum (Figure 2). The patient remained asymptomatic, without signs of biliary obstruction. An endoscopic ultrasound (EUS) was performed and revealed a lobular lesion in the ampulla of Vater. A biopsy of the suspicious site revealed an intestinal adenocarcinoma with a similar immunophenotype to the primary rectal cancer, namely CK7 (−), CD20 (+), CDX2 (+), and DPC4 (+). In order to exclude the presence of distant metastatic disease, an integrated positron emission tomography (PET)/CT scan was performed, which showed increased metabolic activity (standardized uptake values: 10,3) only in the second part of the duodenum. Another multidisciplinary consultation followed where it was decided to proceed with an oncologic resection of the tumor.

The patient underwent a pancreaticoduodenectomy (Whipple procedure). He experienced an uneventful recovery and was discharged on the 16th postoperative day. Histopathological examination revealed an adenocarcinoma in the ampulla of Vater with negative surgical margins (R0 resection) and without lymph nodes infiltration or vascular and perineural invasion; the immunohistochemical examination confirmed the preoperative biopsy's results. Three months after the second operation, the patient started undergoing adjuvant chemotherapy with 12 doses of FOLFOX. Sixteen months following the surgery, he remained disease‐free and received close biannual oncological follow‐up.

## 3 DISCUSSION

Tumor of the ampulla of Vater are quite uncommon, with approximately 4–6 cases per million population.^9^ Moreover, the presence of a secondary tumor located in the ampulla of Vater is extremely rare, with only 33 cases—including the case presented herein‐ reported to date.^7^ The primary malignancy most commonly originates from a renal carcinoma (34%), malignant melanoma (31%) and breast cancer (13%).^7^ The time between the diagnosis of the primary cancer and the detection of the metastatic one varies from synchronous diagnosis to years.^10^

Differential diagnosis between a secondary and second primary ampullary tumor can be quite challenging without the existence of standard criteria. A definitive diagnosis is based on radiological, histological, and immunohistochemical analyses.^7^ Ampullary carcinomas originating from intestinal epithelial cells have variable expression of CK7 and are commonly negative for CK17, contrary to adenocarcinomas arising from the biliary ducts which are typically CK7 and CK17 positive.^10^ Furthermore, intestinal epithelial cells express both CK20 and CDX‐2, an uncommon expression in pancreaticobiliary neoplasms.^11^ The diagnostic accuracy of biopsies for primary ampullary tumors ranges between 47% and 95%.^12^

Multidetector Computed Tomography pancreatography, upper abdomen Magnetic Resonance Imaging and Magnetic Resonance Cholangiopancreatography are considered the standard of care in evaluation and staging of ampullary neoplasms.^13^ Endoscopic retrograde cholangiopancreatography and EUS are valuable tools in the further local evaluation and histological examination of ampullary and periampullary lesions.^13^

The prognosis of patients with a metastatic tumor in the ampulla of Vater is considered poor.^14^ Where no sign of other metastatic lesions exist, surgical removal of the tumor must be considered. Sarocchi et al. reported that half of the patients with a single metastatic tumor in the ampulla of Vater underwent surgical treatment (Whipple procedure reported in 10 cases) and 10 patients underwent endoscopic or surgical ampullectomy.^7^ In addition, a recent meta‐analysis revealed that surgical management of ampullary tumors have an increased rate of complete resection, but a higher risk of complications.^15^

To the best of our knowledge, the present case represents the first reported metastatic tumor in the ampulla of Vater, originating from a rectal adenocarcinoma. The patient underwent a Whipple procedure and 16 months later remains disease‐free. This case underlines the critical role of immunohistochemistry in arriving at a correct diagnosis in order to guide clinical decision‐making.

## CONFLICT OF INTEREST

The authors have no conflict of interest to declare.

## AUTHOR CONTRIBUTIONS

All authors had full access to the data in the study and take responsibility for the integrity of the data and the accuracy of the dataanalysis. *Conceptualization*, A.S., A.K., K.T., C.R.; *Data curation*, A.S., I.‐K.S., A.D., A.K., K.T., G.C.; *Investigation*, A.S., I.‐K.S., A.D., A.K.; *Writing‐original draft*, A.S., I.‐K.S., A.D.; *Writing‐review & editing*, K.T., M.T., G.C., C.R., D.Z.; *Supervision*, M.T., C.R., D.Z.

## ETHICAL STATEMENT

Patient has signed an informed consent form.

## Data Availability

Data available upon reasoning request.
